# West Nile Virus Risk Assessment and the Bridge Vector Paradigm

**DOI:** 10.3201/eid1103.040364

**Published:** 2005-03

**Authors:** A. Marm Kilpatrick, Laura D. Kramer, Scott R. Campbell, E. Oscar Alleyne, Andrew P. Dobson, Peter Daszak

**Affiliations:** * Consortium for Conservation Medicine, Wildlife Trust, Palisades, New York, USA;; † New York State Department of Health, Albany, New York, USA;; ‡ Suffolk County Department of Health Services, Yaphank, New York, USA;; § Rockland County Health Department, Pomona, New York, USA;; ¶ Princeton University, Princeton, New Jersey, USA

**Keywords:** Flavivirus, zoonoses, ecology, Culex, mosquito, disease, transmission

## Abstract

In the northeast United States, control of West Nile virus (WNV) vectors has been unfocused because of a lack of accurate knowledge about the roles different mosquitoes play in WNV transmission. We analyzed the risk posed by 10 species of mosquitoes for transmitting WNV to humans by using a novel risk-assessment measure that combines information on the abundance, infection prevalence, vector competence, and biting behavior of vectors. This analysis suggests that 2 species (*Culex pipiens* L. and *Cx. restuans* Theobald [Diptera: Cilicidae]) not previously considered important in transmitting WNV to humans may be responsible for up to 80% of human WNV infections in this region. This finding suggests that control efforts should be focused on these species which may reduce effects on nontarget wetland organisms. Our risk measure has broad applicability to other regions and diseases and can be adapted for use as a predictive tool of future human WNV infections.

Since its first appearance in North America in 1999, West Nile virus (WNV) has spread across the continent and into Central America. It has infected >17,000 persons and caused >670 deaths (*1**,**2*). Reducing the number of human cases of WNV through vector control depends on efficiently using limited resources (*3*), which requires understanding which vectors are most important in transmitting WNV to humans.

Previous research has suggested that different mosquito species play different roles in spreading WNV. *Culex pipiens* L. and *Cx. restuans* Theobald mosquitoes are thought to be the primary amplification vectors of WNV in birds in the northeastern and north-central United States (*4*) because they are primarily ornithophilic, or bird-biting, are abundant, and have the highest prevalences for WNV in this region (*5*). These species have not been considered important in transmitting WNV to humans because of their feeding habits (*3**,**4*). Instead, other mosquitoes, which take a larger fraction of their blood meals from mammals, are thought to be bridge vectors in transmitting WNV to humans (*3**,**4*). Species that have been proposed as bridge vectors include members of the genera *Aedes* and *Ochleratus* and other *Culex* species (*3**,**4*). However, classification of mosquito species as enzootic or bridge vectors was previously based primarily on qualitative categories and did not incorporate other data that are critical to determining the risk for human infection from each species. In this study, we integrate quantitative information on the abundance, WNV infection prevalence, vector competence, and biting behavior of the most important vectors in the northeast and north-central United States to predict the risk for human infection from each species.

## Materials and Methods

The probability or risk that a species of mosquito will infect a human with WNV can be estimated as Risk = *A* × *F_m_* × *P* × *C_v_*, where *A* is the abundance, *F_m_* is the fraction of blood meals taken from mammals, *P* is the WNV infection prevalence, and *C_v_* is an index of vector competence (the fraction of WNV-infected mosquitoes that will transmit virus in a subsequent bite). Our equation for Risk (capitalized to denote our calculated expression) is an estimate of the relative number of WNV-infectious bites on mammals by each mosquito species. We discuss the data we used for each variable in turn.

We used abundance data from 2 counties near the original 1999 outbreak (Suffolk and Rockland) in New York State during the period 2000–2003, totaling ≈7,195 trap-nights and 378,000 mosquitoes. Mosquitoes were collected by using Centers for Disease Control and Prevention (CDC) light traps baited with CO_2_ (dry ice) from evening until the next morning, which includes the peak activity periods for the mosquitoes considered here. While some mosquitoes are underrepresented in CDC light trap collections (e.g., *Ochleratus trivittatus* [6,7]), baiting traps with CO_2_ and trapping during both dusk and dawn minimizes this bias (*3*).

Mosquitoes from these traps were identified to species with 1 exception; *Cx. pipiens* and *Cx. restuans* adults are difficult to distinguish in the field and are usually counted and submitted for testing after being pooled and labeled as *Cx. pipiens*/*Cx. restuans*. As a result, we present the 2 species as a pair. We averaged their vector competencies and fraction of blood meals from mammalian hosts, which were examined for each species separately by using a molecular identification protocol (*8*) and identification of mosquitoes as larvae to separate the 2 species. Identification of a subset of trapped adult mosquitoes by experienced entomologists (*9*) suggests that although year-to-year variability occurs, these species had approximately equal abundance averaged over the past 4 years (L. Kramer et al., unpub. data). Both species have similar feeding behavior (15%–22% of blood meals come from mammalian hosts, averaging the data weighted by sample size from [9,10] and L. Kramer et al., unpub. data) and breed in similar habitats (containers such as tires, gutters, catch basins, polluted surface pools). As a result, combining these species in our analysis should not detract from our ability to determine which vectors transmit WNV to humans; nor should it alter strategies that should be taken to control vector populations.

To increase the sensitivity of our analyses, we used WNV testing data from all of New York State from 2000 to 2003 to estimate the infection prevalence for each species. However, we only included data from mosquitoes trapped with CDC light traps because prevalences were higher from mosquitoes caught in gravid traps, and these traps primarily capture *Culex* mosquitoes that have already fed at least once. Although the large-scale averaging we performed ignores important spatial and temporal variation, the larger dataset is required to accurately estimate prevalence for species that are rarely infected (i.e., all non-*Culex* species). A correlation analysis of mosquito species' prevalences at the county and state level suggested that the 2 datasets were comparable (Rockland vs. New York State, r = 0.92 and Suffolk vs. New York State, r = 0.97). Mosquitoes were tested for WNV RNA by using reverse transcription–polymerase chain reaction (RT-PCR) (*11*) in groups (pools) of 20 to 50, and infection prevalence of each species is expressed as the minimum infection rate (MIR), where MIR = 1,000 × (pools testing positive for WNV/total number of mosquitoes tested). This calculation assumes that each pool contains only 1 infected mosquito, which is >99% likely for MIRs < 3 and pools of 50. Occasionally, MIRs >3 have been recorded for *Cx. pipiens*/*Cx. restuans* (*5*), which could lead to an underestimate of the true prevalence and Risk for this species pair.

Blood meals from mosquitoes trapped in New York and New Jersey were identified to vertebrate order by using PCR or the heteroduplex method (*9**,**10**,**12*). We calculated the fraction of each species' blood meals that came from mammals as a relative estimate of the probability that the species would feed on humans (*13*). We believe this approximation is valid because identification of mammalian blood meals to the species level showed that all of the species considered here feed on humans (*10**,**14*). In addition, all of the identified mammalian blood meals from *Cx. pipiens* and *Cx. restuans* were from ground-dwelling mammals (9,10, Kramer et al., unpub. data). As a result, we believe that mammalian blood meals identified from *Cx. pipiens* and *Cx. restuans* were not a result of these normally ornithophilic mosquitoes' occasionally biting arboreal mammals. Lastly, recent research showed that North American *Cx. pipiens* are actually hybrids between more ornithophilic *Cx. pipiens* and more opportunistically feeding *Cx. molestus* and *Cx. quinquefasciatus* (*15*), which might help explain their feeding on humans and other mammals.

Finally, previous research has shown that mosquitoes may be infected with WNV (i.e., test positive) but not transmit virus when feeding, at times because the virus is not present in the salivary glands (*4*). The probability that the virus will be transmitted with a bite, given that a mosquito tests positive for WNV, differs among species and has been incorporated into the analysis through the vector competence *C_v_* (see Table for sources). Three species, *Culiseta melanura*, *Ochlerotatus canadensis*, and *Oc. trivittatus*, have not been tested for vector competence. For these species, we used values for their congeners and also present the Risk for these species if their *C_v_* was 1, i.e., if every infected mosquito transmitted the virus when feeding (see Table).

**Table:** Risk of mosquito species transmitting West Nile virus (WNV) to humans

Species	Relative abundance	WNV MIR*	Vector competence† (reference)	Fraction mammal‡	Risk	% Risk
*Aedes vexans*	20.7	0.05	0.17 (16)	0.86 (126)	0.14	4.5
*Coquillettidia perturbans*	11.3	0.01	0.11 (17)	0.83 (191)	0.01	0.5
*Culex pipiens + Cx. restuans*	37.2	0.95	0.38 (16–18)	0.19 (373)	2.52	80.2
*Cx. salinarius*	0.6	0.85	0.36 (17)	0.67 (91)	0.12	3.9
*Culiseta melanura*	5.2	0.17	0.28 (19)§	0.11 (141)	0.03	0.8
*Ochlerotatus canadensis*	14.9	0.00	0.55 (16,18)¶	1.00 (107)	0.00	0.0
*Oc. japonicus*	0.5	0.33	0.93 (16)	0.95 (57)	0.16	5.0
*Oc. sollicitans*	2.0	0.07	0.16 (16)	1.00 (28)	0.02	0.7
*Oc. trivittatus*	7.6	0.05	0.55 (16,18)¶	0.64 (115)	0.14	4.4

*MIR, minimum infection rate. †The fraction of WNV-infected mosquitoes that will transmit virus in a subsequent bite. ‡Number of mosquito blood meals identified in parentheses (9,10, Kramer et al., unpub. data). §Vector competence value taken from study on *Cs. inornata*. Risk increases to 0.09 and 3.0%, assuming a maximum vector competence of 1.0. ¶Genus average used. Risk with a vector competence of 1.0 would be 0 and 0% for *Oc. canadensis* and 0.25 and 8.0% for *Oc. trivittatus*.

## Results

The species-pair *Cx. pipiens* + *Cx. restuans* accounts for >80% of the total Risk, a surrogate for human WNV infections in this region, over this time period (Table). The threat of this species-pair is ≈16 times higher than that for the 4 other important species, *Oc. japonicus*, *Ae. vexans*, *Oc. trivittatus*, and *Cx. salinarius*. This finding is a result of the high WNV prevalence and abundance of this species-pair, which more than compensates for the relatively small fraction of mammalian blood meals of these primarily bird-biting mosquitoes (Table). In contrast, while some of the species previously suggested as important bridge vectors have high infection rates (*Cx. salinarius*), are abundant (*Aedes vexans*, *Oc. canadensis*), or are extremely efficient vectors in the laboratory (*Oc. japonicus*), none make up >5% of the total Risk for human WNV infections.

## Discussion

Integrating 4 important aspects of disease transmission into a single measure of Risk suggests that 2 mosquito species that were previously overlooked as vectors for transmission to humans may in fact be the most important. Current WNV management guidelines (*3*) call for broadly controlling mosquitoes by using both insecticides and water flow management. Our results argue for focusing mosquito control efforts on *Cx. pipiens* and *Cx. restuans*, which primarily breed in a small subset of habitats (tires, gutters, catch basins, polluted surface pools) that are different from those of many other vectors (*20*). This focus could substantially reduce the detrimental effects of mosquito control on nontarget species, especially in wetlands. In addition, focusing control on these habitats and species should improve the effectiveness of control measures and reduce the number of human WNV infections. Finally, reducing the densities of these mosquito species should also decrease transmission of WNV between birds. This management strategy has the dual benefit of decreasing the severity of WNV epidemics in birds and the subsequent spillover to mammals.

These results should be placed within their proper spatial and geographic context. The most important vectors for transmitting WNV to humans in other regions of the United States are likely to be different. *Cx. quinquefasciatus* and *Cx. nigripalpus* are the predominant vectors of WNV between birds in the southeastern United States (*17**,**21*), and *Cx. tarsalis* and *Cx. quinquefasciatus* play this role in much of the western United States (*19*). Broad feeding habits, host switching from birds to mammals in the fall, or both (*22**–**24*) make these 3 species likely to also be the dominant vectors in transmitting WNV to humans in these regions. Similarly, while our analysis of vectors in the northeastern United States determined the most important vectors for human WNV infections by averaging over several years and a multicounty scale, other vectors may be more important on a local scale (e.g., *Cx. salinarius* near a salt marsh [25]) or during portions of the transmission season. Our results should be verified at smaller temporal and spatial scales because averaging over data in which abundance and infection rates negatively covary can produce biased results (*26*).

The validity of our conclusions rests on the assumptions we have made and the data on which they are based. Of primary importance is the relative number of feedings of *Cx. pipiens* and *Cx. restuans* on humans, which is based on abundance estimates generated by using CO_2_-baited CDC light traps and mosquito blood meals identified from mammalian hosts (as well as a small number from humans). Recent work by Gingrich and Casillas (*25*) strengthens our results and suggests that feedings by *Cx. pipiens* on humans may be more common than was previously thought. These researchers compared the landing rates of mosquitoes on humans (which were then captured with an aspirator and identified) with the abundance of mosquitoes trapped with CO_2_-baited CDC light traps at 4 sites in Delaware. When data from their Table 1 were used, the ratio of mosquitoes caught after landing on a human to those caught by using CO_2_-baited CDC light traps is 0.36 for *Cx. pipiens*, 0.40 for *Cx. salinarius*, and 0.07 for *Ae. vexans* (25, Table). This finding suggests that in terms of feeding on humans, *Cx. pipiens* are relatively underrepresented by CO_2_-baited CDC light traps compared to *Ae. vexans*, which implies that *Ae. vexans* may be less important and *Cx. pipiens* more important than our analyses have shown. Of the species considered in both our study and that of Gingritch and Casillas, only *Oc. canadensis* is relatively underrepresented by CDC light traps compared to *Cx. pipiens*, with a human landings to CDC light trap ratio of 1.31. However, this species is rarely infected with WNV and does not represent an important vector for transmitting WNV to humans (Table).

One strength of our Risk measure is that it can be applied to other locations and at other scales simply by applying the risk equation to data from the desired scale and region if analyzed appropriately (*26*). In addition, the Risk equation can be used as a predictive index to forecast the relative number of future human WNV infections, which could be useful for short-term planning and resource allocation. The sum of the Risk equation over all (i = 1 to n) mosquito species multiplied by human population density in the area considered should estimate the number of short-term future human WNV infections:

Predicted no. of human WNV infections = (human density) × _
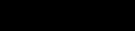
_

We are currently testing the usefulness of this index in predicting the relative number of human WNV infections between locations and over the mosquito season.
